# Microbial communities on fish eggs from *Acanthopagrus schlegelii* and *Halichoeres nigrescens* at the XuWen coral reef in the Gulf of Tonkin

**DOI:** 10.7717/peerj.8517

**Published:** 2020-02-07

**Authors:** Shijie Bai, Gang Hou

**Affiliations:** 1 Deep Sea Science Division, Institute of Deep Sea Science and Engineering, Chinese Academy of Sciences, Sanya, Hainan, China; 2 Guangdong Ocean University, Zhanjiang, Guangdong, China

**Keywords:** Microbial community, Fish eggs, *Acanthopagrus schlegelii*, *Halichoeres nigrescens*, Coral reef

## Abstract

Coral reefs are an important part of the ocean ecosystem and are a vital spawning ground for marine fish. Microorganisms are abundant in this environment and play a key role in the growth and development of host species. Many studies have investigated the microbial communities of fish with a focus on the intestinal microbiome of laboratory-reared adult fish. Little is known about the relationship between fish eggs and their microorganisms, especially as microbial communities relate to wild fish eggs in coral reefs. In this study, we analyzed the microbial communities of two species of coral fish eggs, *Acanthopagrus schlegelii* and *Halichoeres nigrescens*, using 16S rRNA gene amplicon sequencing technology. *Pseudomonas*, *Archromobacter,* and *Serratia* were the main bacterial genera associated with these fish eggs and are known to be bacteria with potentially pathogenic and spoilage effects. The microbial community structures of *Acanthopagrus schlegelii* and *Halichoeres nigrescens* eggs were separated based on the 30 most abundant operational taxonomic units (OTUs). Principal coordinate analysis (PCoA) and non-metric multidimensional scaling analysis (NMDS) further confirmed that the microbial communities of coral fish eggs differ by species, which may be due to host selection. A functional prediction of the microbial communities indicated that most of the microbial communities were chemoheterotrophic and involved in nitrogen cycling. Our results showed that the microbial communities of coral fish eggs were distinct by species and that key microorganisms were potentially pathogenic, leading to the spoilage of fish eggs, high mortality, and low incubation rates. This study provided new insights for understanding the relationship between microorganisms and wild fish eggs.

## Introduction

Coral reef ecosystems play crucial roles in the primary productivity and biological diversity of the ocean (Moberg & Folke, 1999). An abundance of microorganisms and marine animals live in the coral reef area, using it as a place to spawn and nurse their young (Doherty, Planes & Mather, 1995; Froukh & Kochzius, 2007). The tropical reefs account for 0.1% of the total ocean area but harbor over 6,300 species of fish, representing half of marine fish species (Parravicini et al., 2013).

Microorganisms are a key component of marine ecosystems, driving nutrient cycling and promoting the stability of the ecosystem (Hutchins & Fu, 2017). Bacteria have extensive interactions with fish, especially in the gut. The microbial communities in the gastrointestinal tract can be categorized into symbiotic and pathogenic microorganisms. Several phyla have been shown to be dominant in the fish gut including *Proteobacteria*, *Firmicutes*, *Bacteroidetes*, *Actinobacteria,* and *Fusobacteria* (Butt & Volkoff, 2019). However, there are distinct microbial communities found among the gastrointestinal tracts of different fish species and among their different life stages (Larsen, Mohammed & Arias, 2014; Yan et al., 2016; Ye et al., 2016). Many efforts have been made to identify the microbial communities of the fish gut, but most have focused on adult fish and a few juveniles taken from cultured samples rather than from a natural environment. Few studies have focused on the microbial communities found in fish eggs (Liu et al., 2014; Wilkins, Fumagalli & Wedekind, 2016; Míguez & Combarro, 2003; Hansen & Olafsen, 1989; Roalkvam et al., 2019), especially those in the wild. The healthy growth and development of eggs is crucial for fisheries. For instance, fish eggs colonized by pathogenic bacteria may have a decreased hatching rate, and reduced output of larvae and juveniles, which ultimately affects the resulting fish population. Therefore, in order to better protect fishery resources, the microbial communities of fish eggs should be studied to determine their specific composition and impact on the eggs.

The Xuwen Coral Reef National Nature Reserve, located in the Guangdong province of China, is the largest and best preserved fringing reef on the coast of China. The sea area of the reef is an important spawning ground for fish in the Gulf of Tonkin, making it an ideal site in which to study the dynamics of the microbial communities among the eggs of different fish species. We investigated the microbial communities of two different species of coral fish eggs to determine whether the microbial communities differed by fish egg species. The microbial community composition of the eggs of different fish species and the predicted metabolic functions of the corresponding microbial communities were also studied.

## Materials & Methods

### Sample collection

**Figure 1:**
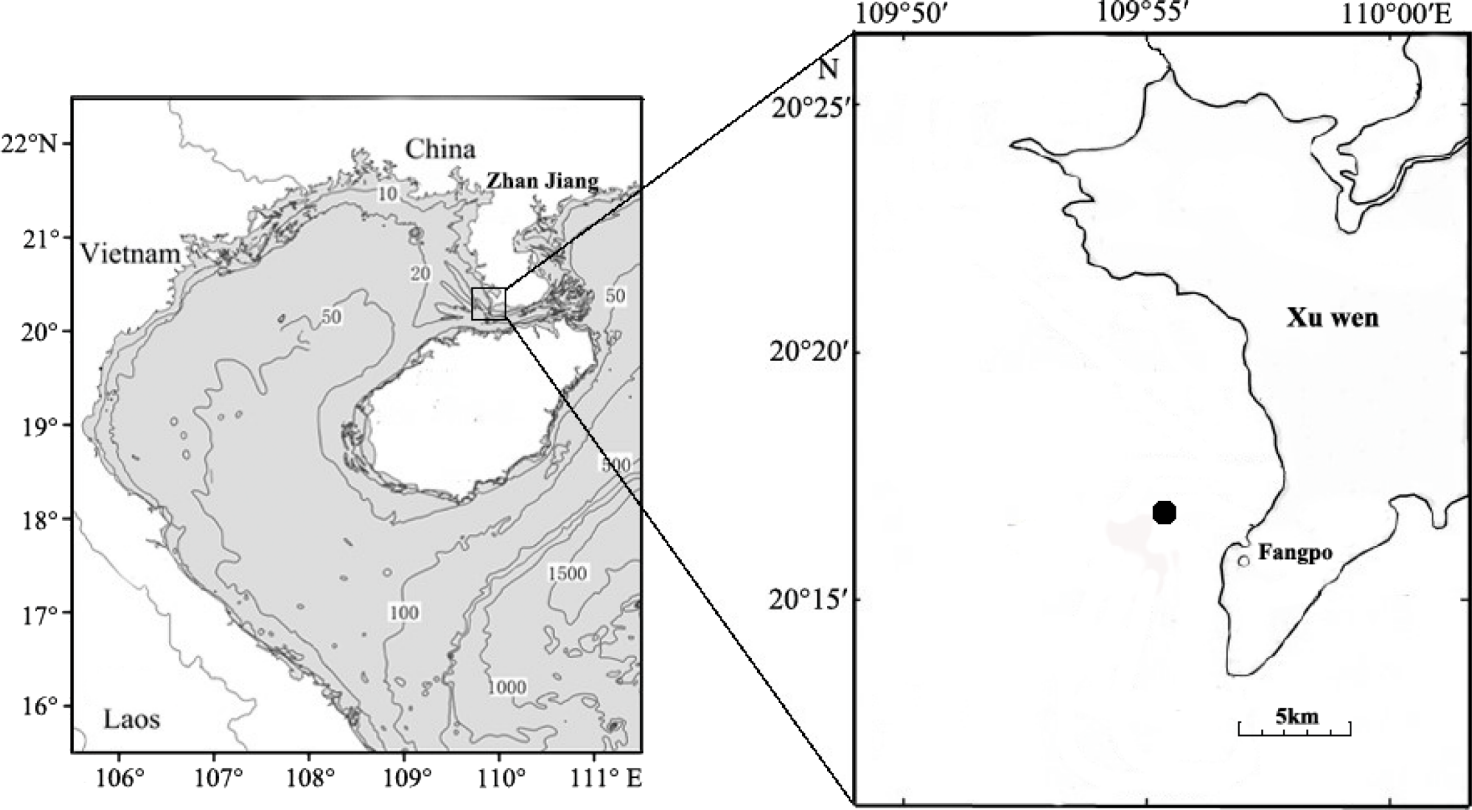
Sampling locations for fish eggs on the Xuwen coral reef.

Fish eggs were collected on December 20th, 2018 at Fangpo Station (109°55′49.08″E, 20°14′11.04″N), in the Xuwen Coral Reef National Nature Reserve, Zhanjiang City, in the Guangdong province of China (Fig. 1). Fish eggs were collected with an 80 cm diameter zooplankton net equipped with a 2.7 m long net with 0.505 mm mesh. The cod-end container had a mesh size of 400 um. Nets were dragged for 15 min on horizontal trawls with a speed of 0.5–1.0 knot/hour. All samples were stored in liquid nitrogen and transferred to the laboratory.

### DNA extraction and COI gene sequencing

DNA was extracted from the fish eggs using the AxyPrep™ Multisource Genomic DNA Miniprep Kit (Axygen^®^), according to the manufacturer’s instructions. The quality of the genomic DNA was checked with a NanoDrop spectrophotometer and Qubit 3.0. The COI (cytochrome C oxidase subunit 1 gene) sequences (∼648 bp) were amplified and sequenced using the universal primers FishF1 and FishR1 (Ward et al., 2005) The polymerase chain reaction (PCR) contained approximately 100 ng of template DNA, 1 µl of each primer (10 pmol), 4 µl of 10 × reaction buffer, 1.2 ul of dNTPs (10 mM each), and 5.0 U of Taq DNA polymerase (FSTM DNA polymerase, P1071, Dongsheng Biotech Co.,Ltd, Guangzhou, China) in a total volume of 40 ul. PCR was conducted under an initial denaturation cycle of 95 °C for 2 min, 35 cycles at 94 °C for 30 s, 51 °C for 30 s, 72 °C for 1 min, and a final extension at 72 °C for 10 min. The product of the PCR reactions was sequenced bidirectionally on an ABI 3730 XL DNA system following the manufacturer’s protocol (Perkin-Elmer Applied Biosystems). Tracer files and assembled sequences were checked using the SEQMAN in LASERGENE version 7.0 (DNASTAR Inc., Madison, WI, USA). High-quality sequences were aligned and manually edited using MEGA v6.0 (Tamura et al., 2013). Fish eggs were identified through Blast searches in BOLD. Sequences with more than 99% similarity and a more than 2% divergence threshold between sequences and the nearest neighbor species were tagged with the taxa name of the fish species (http://www.boldsystems.org/). The local fish fauna list and DNA barcode library were used in combination with BOLD to confirm the fish egg species when the above criteria were not met (Hou et al., 2018).

### 16S rRNA library preparation and sequencing

The results of the fish egg identification yielded nine DNA samples from *Acanthopagrus schlegelii* and seven DNA samples from *Halichoeres nigrescens*, which were used to construct 16S rRNA V4 sequencing amplicons using the primer pair 515f Modified and 806r Modified (Walters et al., 2015). PCR cycling conditions were as follows: denaturation at 95  °C for 3 min, 27 cycles of 95 °C for 30 s, 55 °C for 30 s, and 72 °C for 45 s, and a final extension at 72 °C for 10 min. Triplicate PCR amplicons were mixed after purification using a TaKaRa purification kit (TaKaRa, Japan). Libraries were then generated using the TruSeq DNA sample preparation kit from Illumina (Illumina, San Diego, CA, USA), following the manufacturer’s instructions. The libraries were sequenced using a MiSeq platform (Illumina) in a paired-end 250 bp sequence read run at MajorBio Co. Ltd. (Shanghai, China).

### Data processing

Raw reads were categorized according to their barcodes and forward and reverse primers, allowing for one mismatch each. Paired end reads of sufficient length were combined with at least a 30 bp overlap into full-length sequences by FLASH program version 1.2.8 (Magoč & Salzberg, 2011). The average fragment length was 253 bp. The Btrim program version 0.2.0 was applied to filter out low quality sequences. The quality score was set to >20 with a 5-base window size as a standard; any sequences containing N’s or that were <200 bp were discarded. The sequences with lengths of 245 bp to 260 bp were kept as targeted sequences (Kong, 2011). UPARSE (Edgar, 2013) was used to remove chimeras and to cluster sequences into 97% identical operational taxonomy units (OTUs); singletons were kept for further analysis. A representative sequence from each OTU was selected for taxonomic annotation by comparison to the full SILVA 128 database (Quast et al., 2013) which included bacterial, archaeal, and eukaryotic sequences. The OTU table was randomly subsampled to normalize the reads of each sample. Raw sequencing reads from all samples can be openly accessed on the NCBI database (http://www.ncbi.nlm.nih.gov/) under BioProject accession number PRJNA560485.

### Statistical analysis

The diversity of the microbial community from *A. schlegelii* and *H. nigrescens* was determined by the statistical analysis of the alpha diversity indices. The Shannon and Inverse Simpson indexes were calculated using the vegan package in R language version 3.4.3 (R Core Team, 2018). The rarefaction curve and Chao1 values (Chao, 1984) were generated by the Mothur program (Schloss et al., 2009). PyNAST was used to align the selected representative OTU of all samples (Caporaso et al., 2010), the tree file was obtained from FastTree (Price, Dehal & Arkin, 2009), and the Phylogenetic diversity (PD) was calculated with the Picante package in R (Kembel et al., 2010). The random forest analysis was conducted using the randomForest package in R. Principal coordinate analysis (PCoA) and non-metric multidimensional scaling (NMDS) were generated to compare the differences within the microbial community structure. The multi response permutation procedure (MRPP), a one-way ordered analysis of similarity (ANOSIM) and permutational multivariate analysis of variance (PERMANOVA) were used to determine whether there were any dissimilarities in the microbial communities of *A. schlegelii* and *H. nigrescens* (*P* > 0.05).

### Predictive functional profiling of microbial communities

Functional metagenomes of the microbes from *A. schlegelii* and *H. nigrescens* eggs were predicted based on the 16S rRNA sequencing data by PICRUSt (Langille et al., 2013). The OTU table was generated against the GreenGenes v13.5 database at a 97% sequence similarity using the closed reference OTU-picking method. A virtual metagenome of KEGG Ortholog abundances was produced for each sample in the given OTU table. A text file was generated with the accuracy metrics for the predicted metagenome and the final metagenomic functional predictions based on the KEGG pathways at classification levels 2 and 3 were generated. The Functional Annotation of Prokaryotic Taxa (FAPROTAX) (Louca, Parfrey & Doebeli, 2016) was used to convert taxonomic microbial community profiles into putative functional profiles based on taxa identified in the sample. FAPROTAX defines functional groups in terms of the taxa (e.g., species or genera) affiliated with each functional group. These affiliations are typically based on peer-reviewed literature, such as announcements of cultured representatives.

## Results

### Fish egg identification

**Figure 2:**
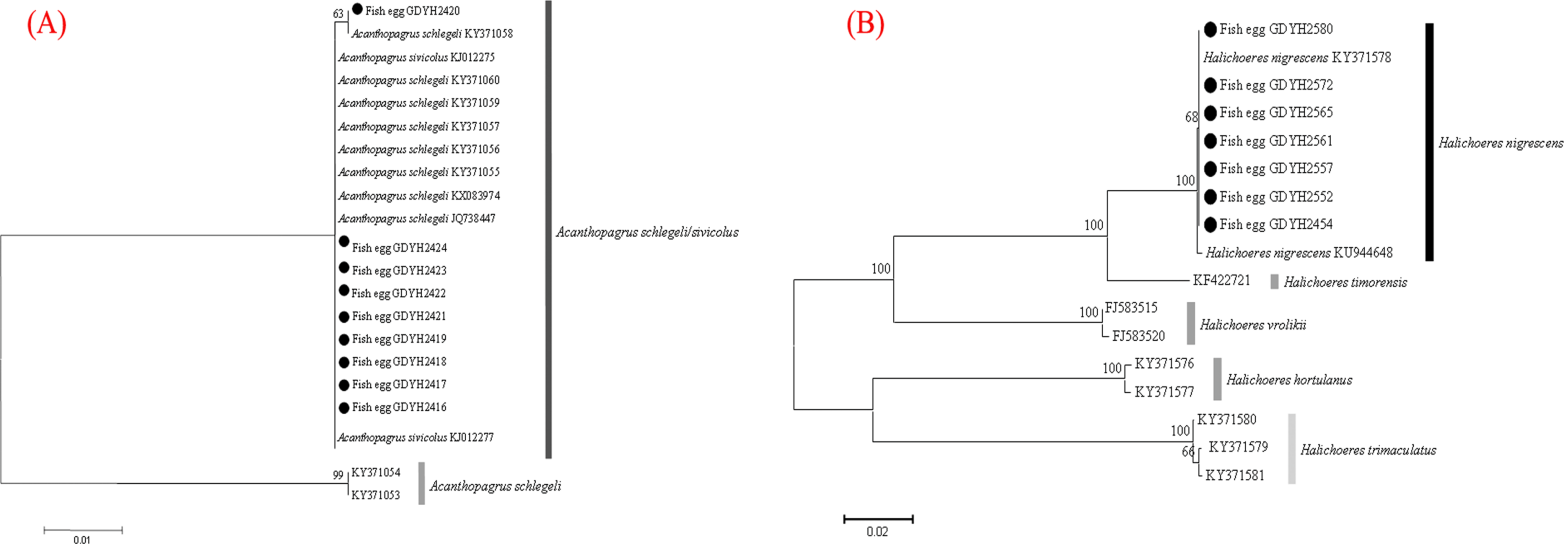
Egg identification of fish species *Acanthopagrus schlegeli* (A) and *Halichoeres nigrescens* (B) based on Neibour-joining tree combined with downloaded adult fish COI sequences in NCBI.

**Figure 3:**
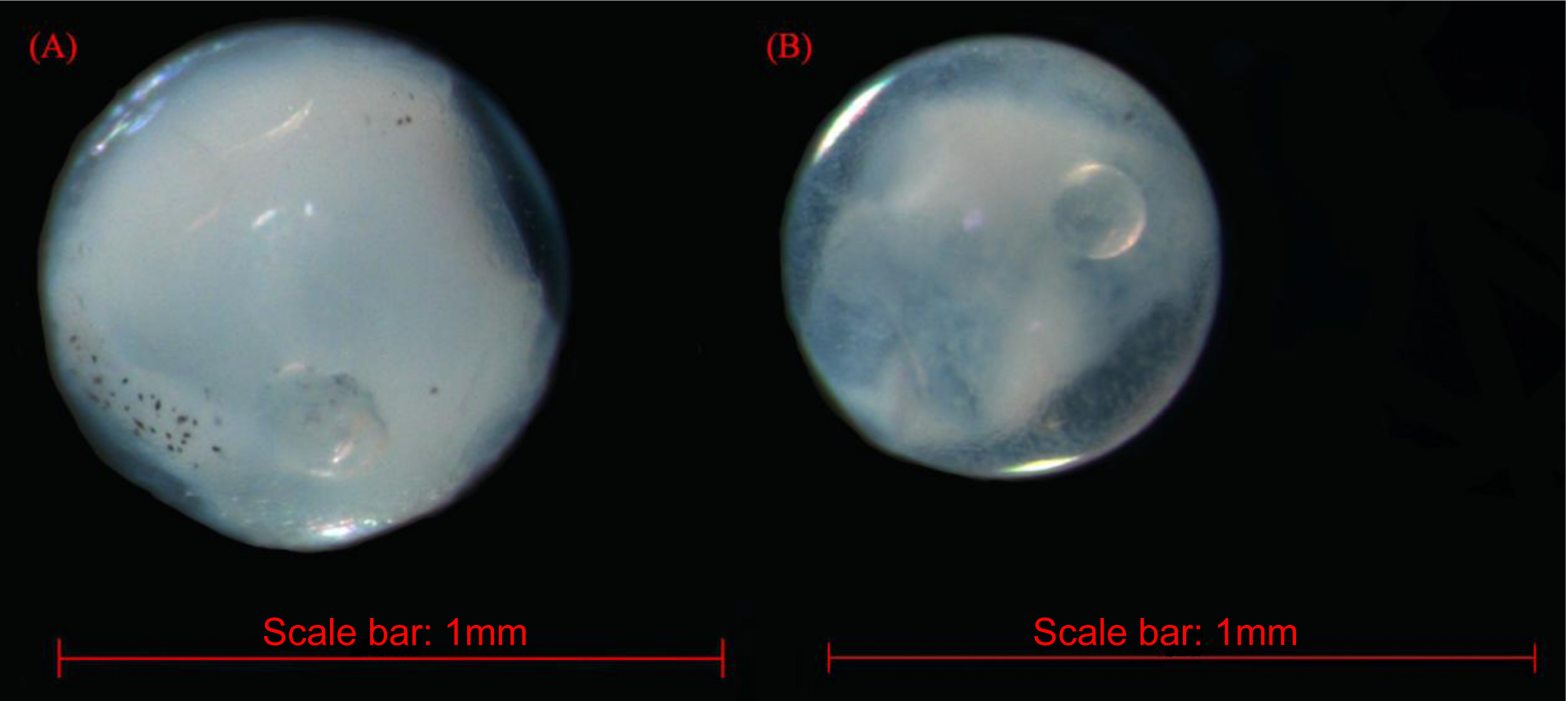
The images of *Acanthopagrus schlegelii* (A) and *Halichoeres nigrescens* (B) eggs; the chosen fish eggs were initially photographed using Zeiss microscope (Axioplan 2 imaging E).

The fish eggs were classified into two morphological types through microscopic examination during sampling. DNA was extracted from 16 individuals and the samples were all successfully amplified and assigned to high quality sequences with no ambiguities noted. The 16 eggs belonged to 3 haplotypes, including haplotype 1 (8 individuals), haplotype 2 (1 individual), and haplotype 3 (7 individuals). Using a rigorous species delimitation (99% of similarity and 1% of genetic divergence among species) haplotypes 1 and 2 matched two fish species: *Acanthopagrus schlegeli* and *A. sivicolus*, and haplotype 3 matched the unique fish species, *Halichoeres nigrescens* (see supporting information 1). Haplotypes 1 and 2 had one lineage in the neighbor-joining (NJ) tree based on local fish COI sequences from a DNA barcode library from a previous study (Hou et al., 2018) (Fig. 2). *A. sivicolus* was not reported in the fish fauna list of the local and adjacent sea areas and the eggs were identified as *A. schlegeli.* The diameter of *A. schlegelii* eggs (haplotypes 1, 2) was 0.90 ± 0.04 mm, and that of *H. nigrescens* eggs was 0.65 ± 0.02 mm (haplotype 3) (Fig. 3).

### Sequencing statistics and microbial diversity

**Figure 4:**
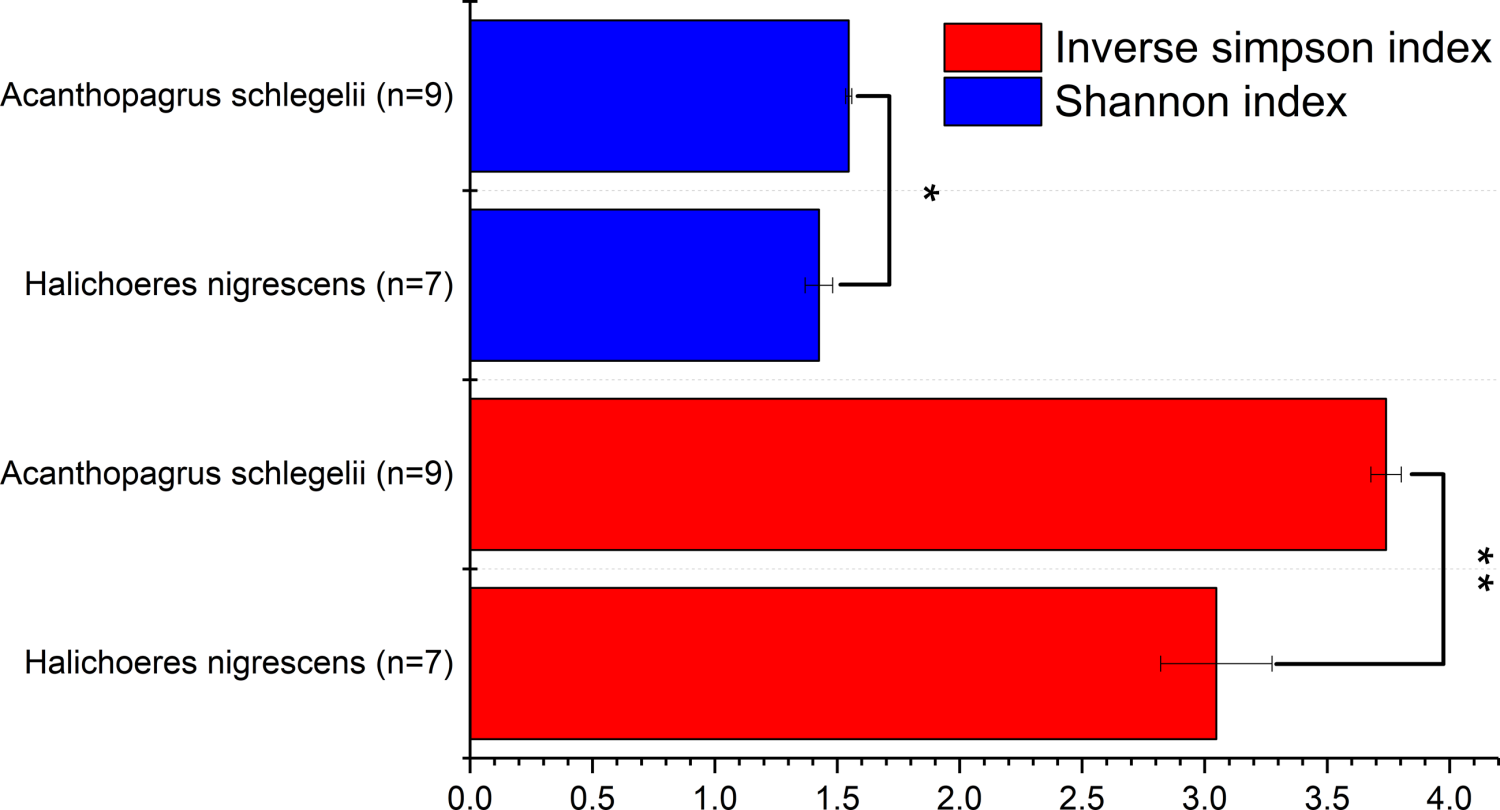
Comparisons of two alpha diversity indexes, Shannon index and Inverse Simpson index. The value is the mean of the indices within each group(*Acanthopagrus schlegelii* group, *n* = 9; *Halichoeres nigrescens* group, *n* = 7), error bars stand for standard errors(SE), *p < 0.05; **p < 0.01; ***p < 0.001 based on Student’s *t* test.

To determine the diversity of the microbial communities of the eggs from *A. schlegelii* and *H. nigrescens*, the V4 region of the 16S rRNA gene was amplified and sequenced using high throughput sequencing. A total of 946,202 sequences were classified into 16 egg samples after being assessed for quality. An average of 59,138 ± 11,817 sequences per sample were obtained and the rarefaction curves indicated a sufficient number of samples (Fig S1). We randomly subsampled 35,215 sequences per sample for the next analyses of microbial diversity, composition, and structure. The alpha diversity of the microbial communities from the eggs of *A. schlegelii* and *H. nigrescens* were calculated. The results of the Shannon and Inverse Simpson indexes indicated that the *α*-diversity of *A. schlegelii* eggs was higher than that of *H. nigrescens* eggs. There was a significant difference indicated by both indices, with *p* = 0.033 for the Shannon index, and *p* = 0.005 for the Inverse Simpson index (Fig. 4).

### Structure and composition of microbial communities

**Figure 5:**
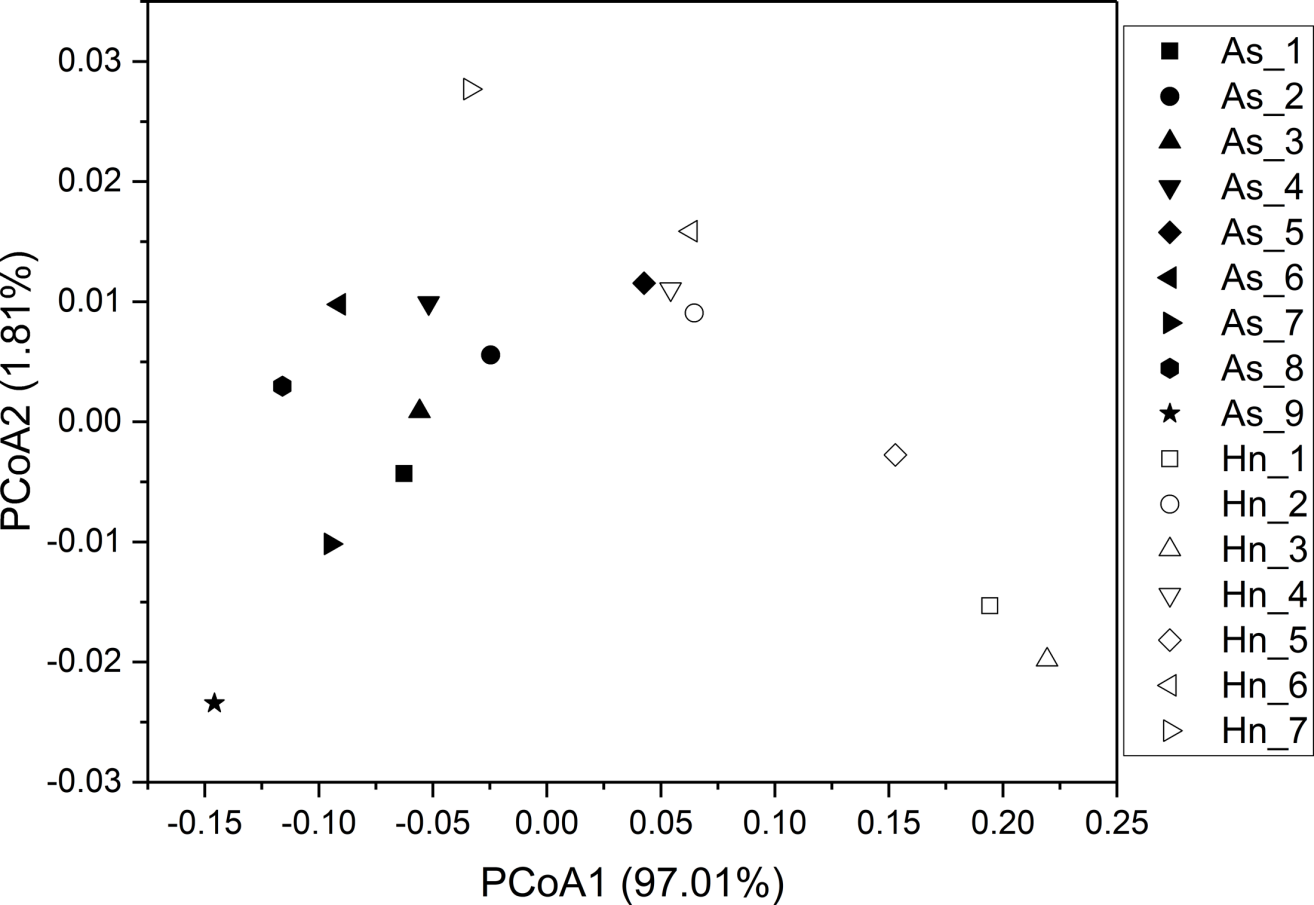
Principal coordinate analysis (PCoA) of microbial communities from *Acanthopagrus schlegelii* (As) and *Halichoeres nigrescens* (Hn) eggs based on weighted UniFrac distance of detected OTUs.

**Figure 6:**
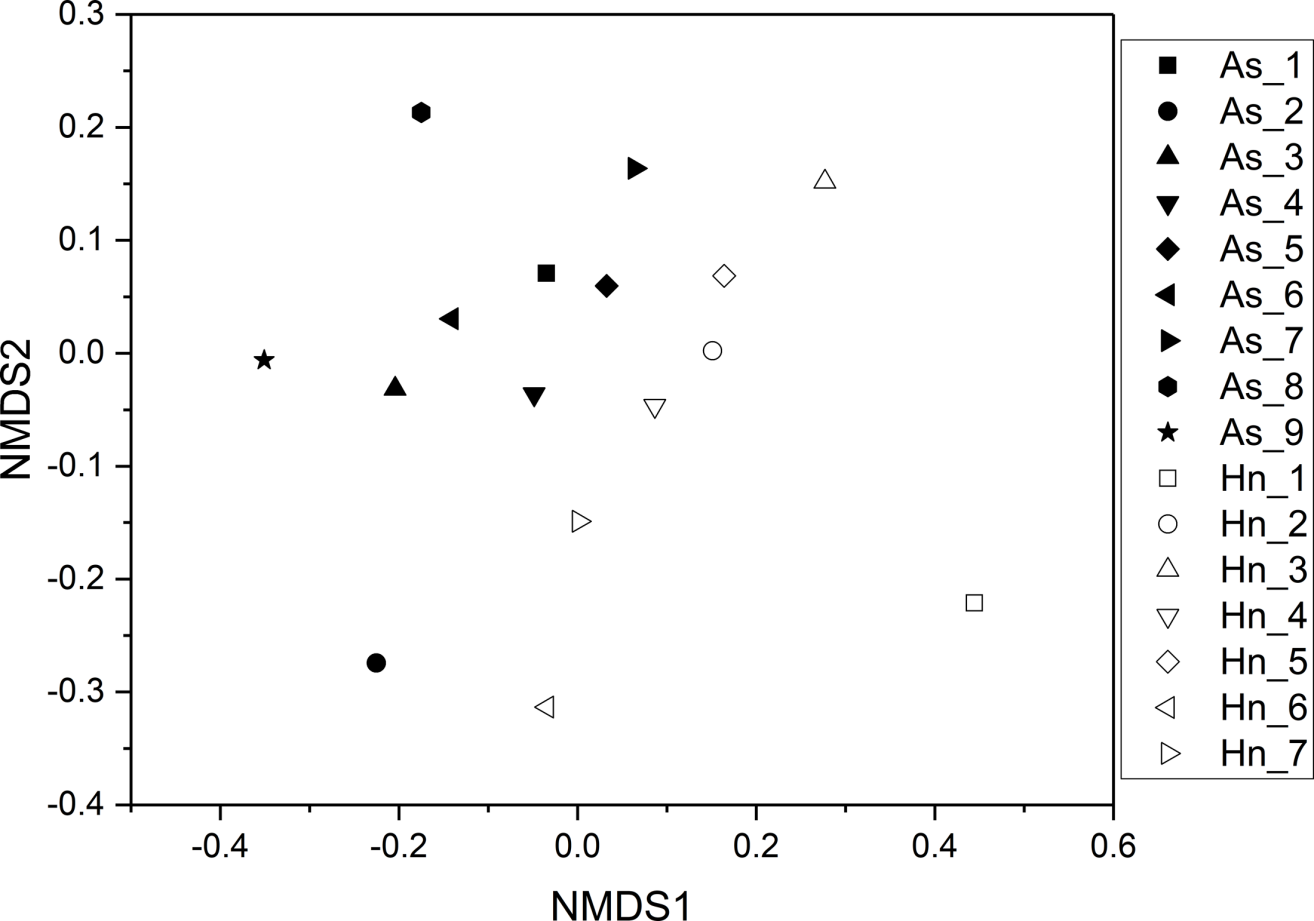
Non-metric multidimensional scaling analysis (NMDS) with Bray–Curtis distance matrix to visualize the structure of microbial community from *Acanthopagrus schlegelii* (As) and *Halichoeres nigrescens* (Hn) eggs.

ß-diversity-based statistical tools, such as the principal coordinate analysis (PCoA) and the nonmetric multidimensional scaling analysis (NMDS) were applied to test the microbial community structure of different species of fish eggs. PCoA and NMDS showed that the microbial community structures were distinct from one another (Figs. 5 and 6), suggesting that different species of fish eggs harbored different microbial communities. A significant difference was observed between *A. schlegelii* and *H. nigrescens* eggs after testing with the multi response permutation procedure (MRPP), one-way ordered analysis of similarity (ANOSIM), and permutational multivariate analysis of variance (PERMANOVA) (Table 1).

**Table 1:** Dissimilarity tests of microbial communities of different fish species eggs based on Jaccard and Bray–Curtis distance.

PERMANOVA	Jaccard		Bray–Curtis	
	*F*	*p*	*F*	*p*
Group (As and Hn)	1.3434	0.003(^**^)	18.2087	0.001(^***^)
Group (As and Hn)	Jaccard		Bray–Curtis	
	*p*		*p*	
MRPP	0.002(^**^)		0.001(^***^)	
ANOSIM	0.003(^**^)		0.002(^**^)	

**Notes.**

* Difference is significant at 0.05 level.

** Difference is significant at 0.01 level.

*** Difference is significant at 0.001 level.

As refer to *Acanthopagrus schlegelii*, Hn refer to *Halichoeres nigrescens*.

**Figure 7:**
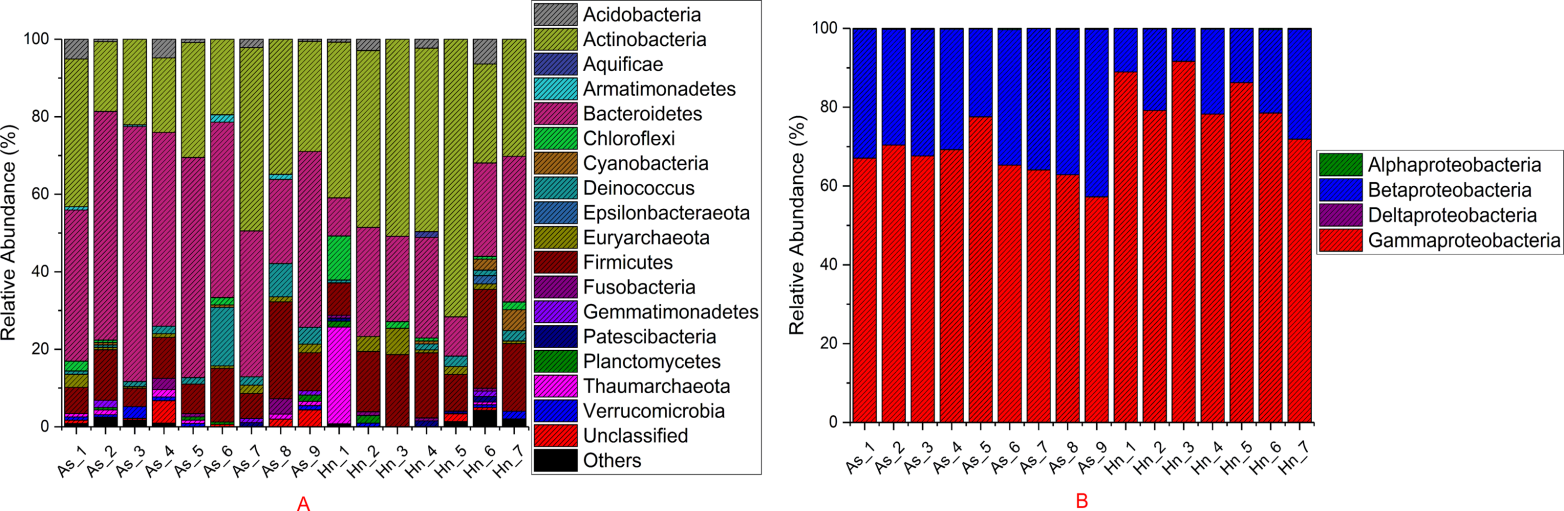
Stacked bar chart showing relative abundance of microbial composition *Acanthopagrus schlegelii* (As) and *Halichoeres nigrescens* (Hn) eggs at the phyla except the phylum of Proteobacteria (A) and the classes level of Proteobacteria (B).

**Figure 8:**
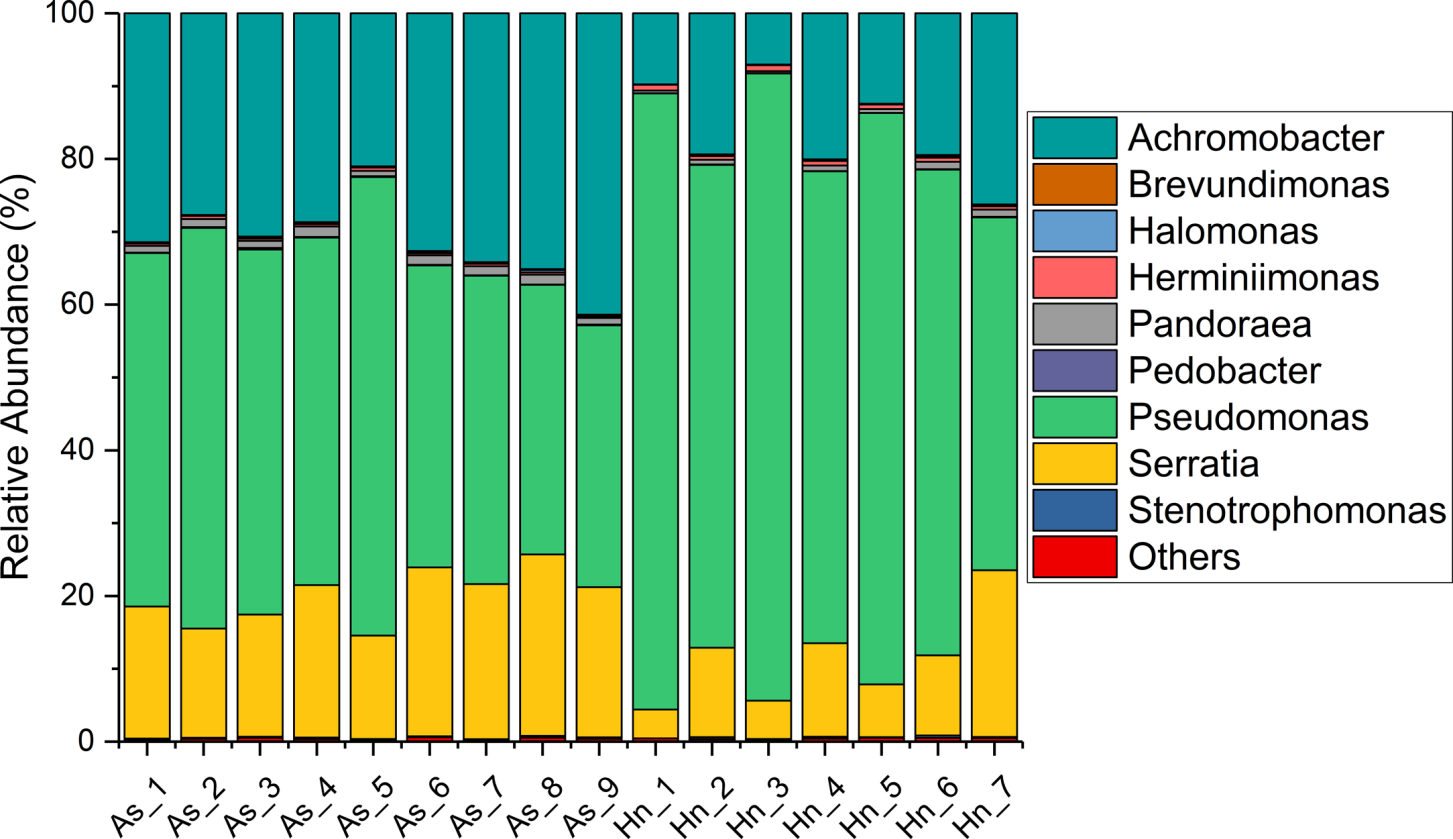
Stacked bar chart showing relative abundance of microbial composition of *Acanthopagrus schlegelii* (As) and *Halichoeres nigrescens* (Hn) eggs at the genera level.

**Figure 9:**
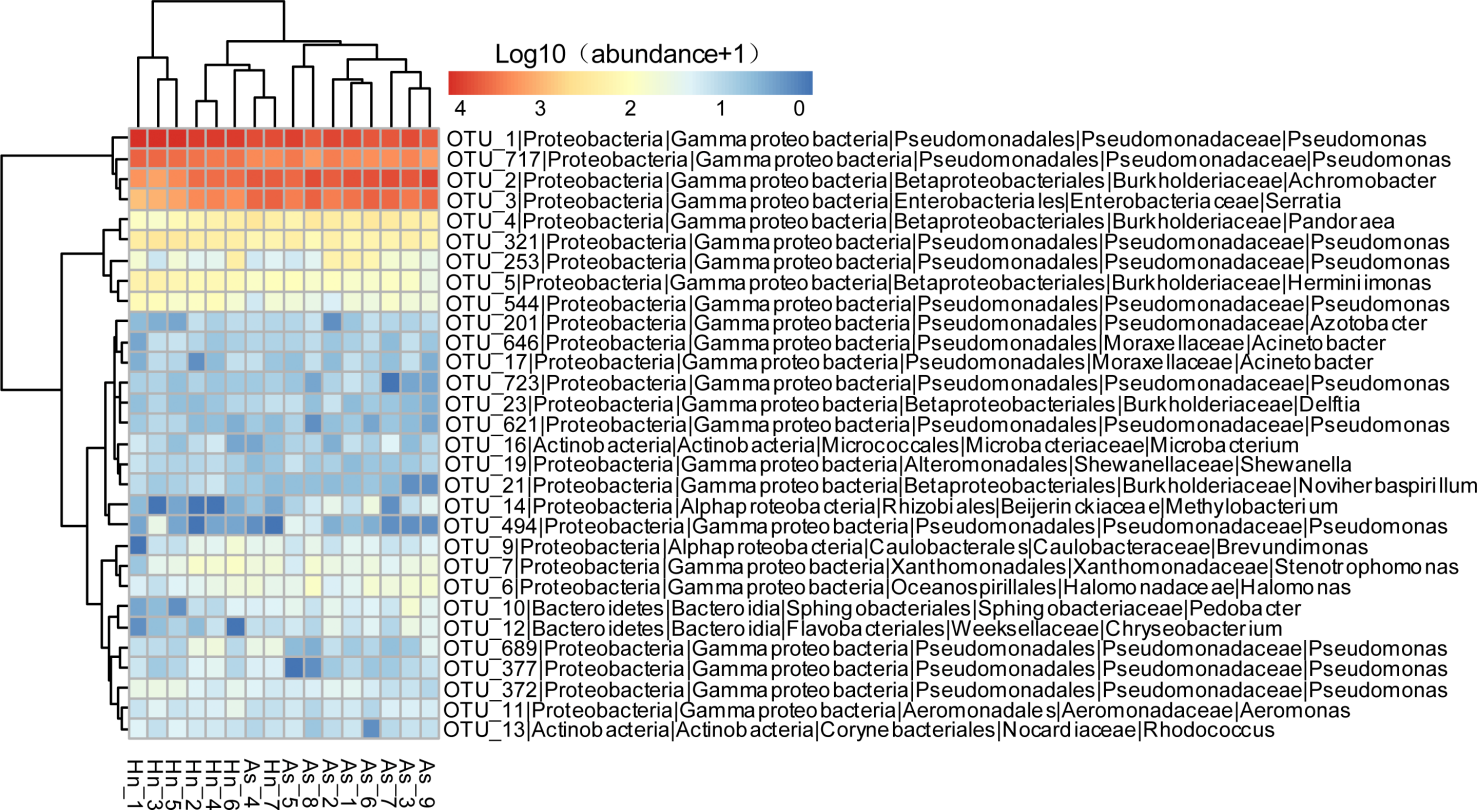
Abundance of the 30 most abundant OTUs of *Acanthopagrus schlegelii* (As) and *Halichoeres nigrescens* (Hn) eggs. Microbial abundance was scaled with log transformation in the heatmap.

The relative abundance of microorganisms was shown at the phylum, class, and genus levels with a similarity of 97% for OTU classification in order to provide detailed information about the composition of the microbial communities (Figs. 7 and 8). The dominant bacterial classes were Gamma- and Betaproteobacteria, which comprised 56.9% to 91.5% and 11.0% to 42.3% of all samples, respectively. Furthermore, the genera level was dominated by *Pseudomonas* (35.8% 85.9%), *Archromobacter* (7.0% to 41.1%), and *Serratia* (4.0% to 24.8%) bacteria, accounting for 97.6% to 98.1% of sequences in all samples. The relative abundances of *Achromobacter* and *Serratia* from *A. schlegelii* eggs were significantly higher than those from *H. nigrescens* eggs (*p* = 0.0002, and *p* = 0.004, respectively, *t* test). However, the relative abundance of *Pseudomonas* from *H. nigrescens* eggs was significantly higher than that of *A. schlegelii* eggs (*P* = 0.0006, *t* test). In addition, the 30 most abundant OTUs were analyzed using the random forest package in R. The results of random forest analysis based on the 30 most abundant OTUs showed the overall out-of-bag (OOB) error was 12.5%, the class error of *A. schlegelii* was 11.11%, and *H. nigrescens* eggs was 14.29%, which suggests that the 30 most abundant OTUs could represent the vast majority of microbial communities in the eggs of two different species of fish. The heatmap based on the 30 most abundant OTUs indicated that different species of fish eggs could harbor distinct microbial communities (Fig. 9).

### Predicted metabolic potential by PICRUSt and FAPROTAX

**Figure 10:**
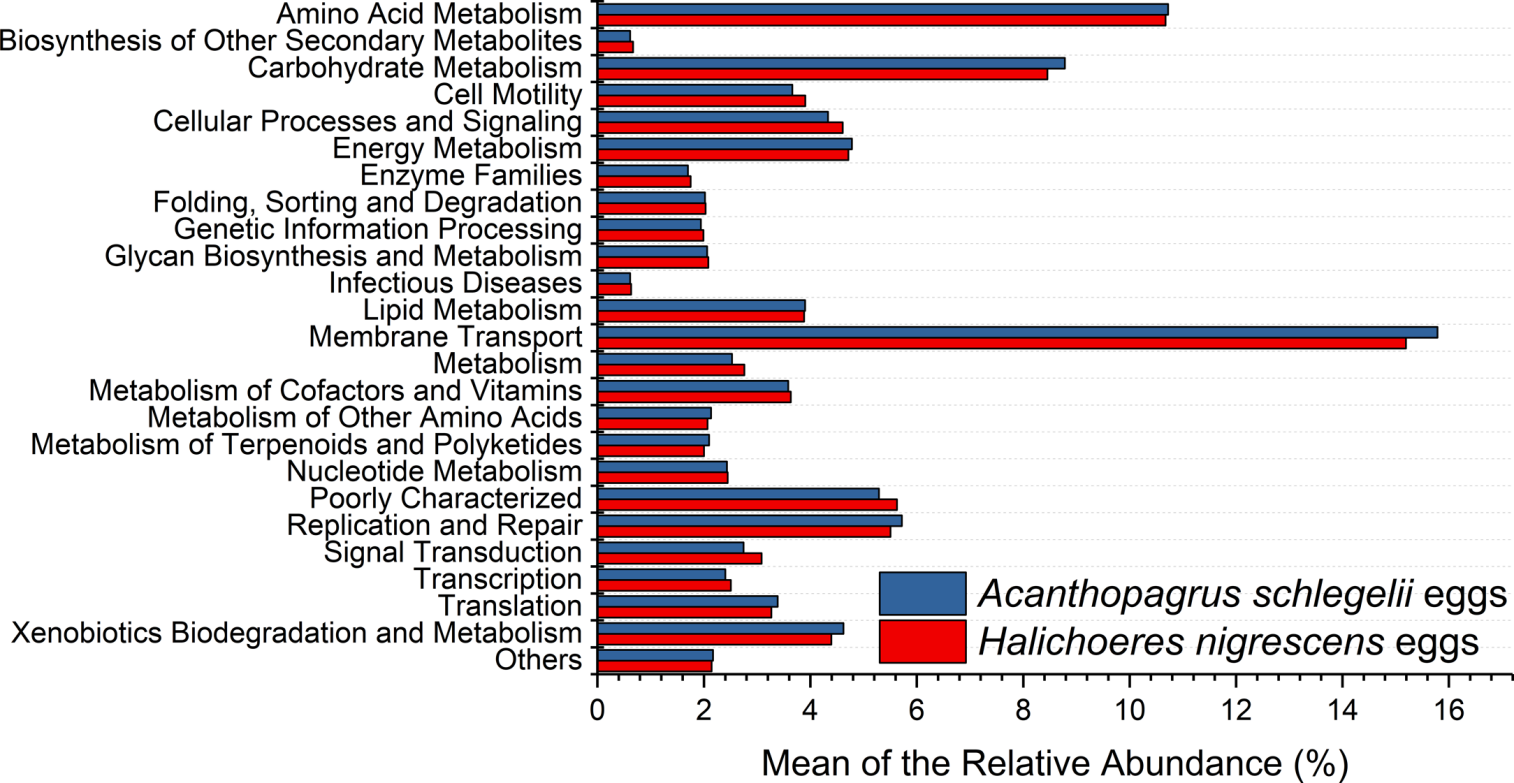
Stacked bar chart showing Mean of the relative abundance of predicted metabolic potential of microbes from *Acanthopagrus schlegelii* and *Halichoeres nigrescens* eggs, as predicted by PICRUSt.

**Figure 11:**
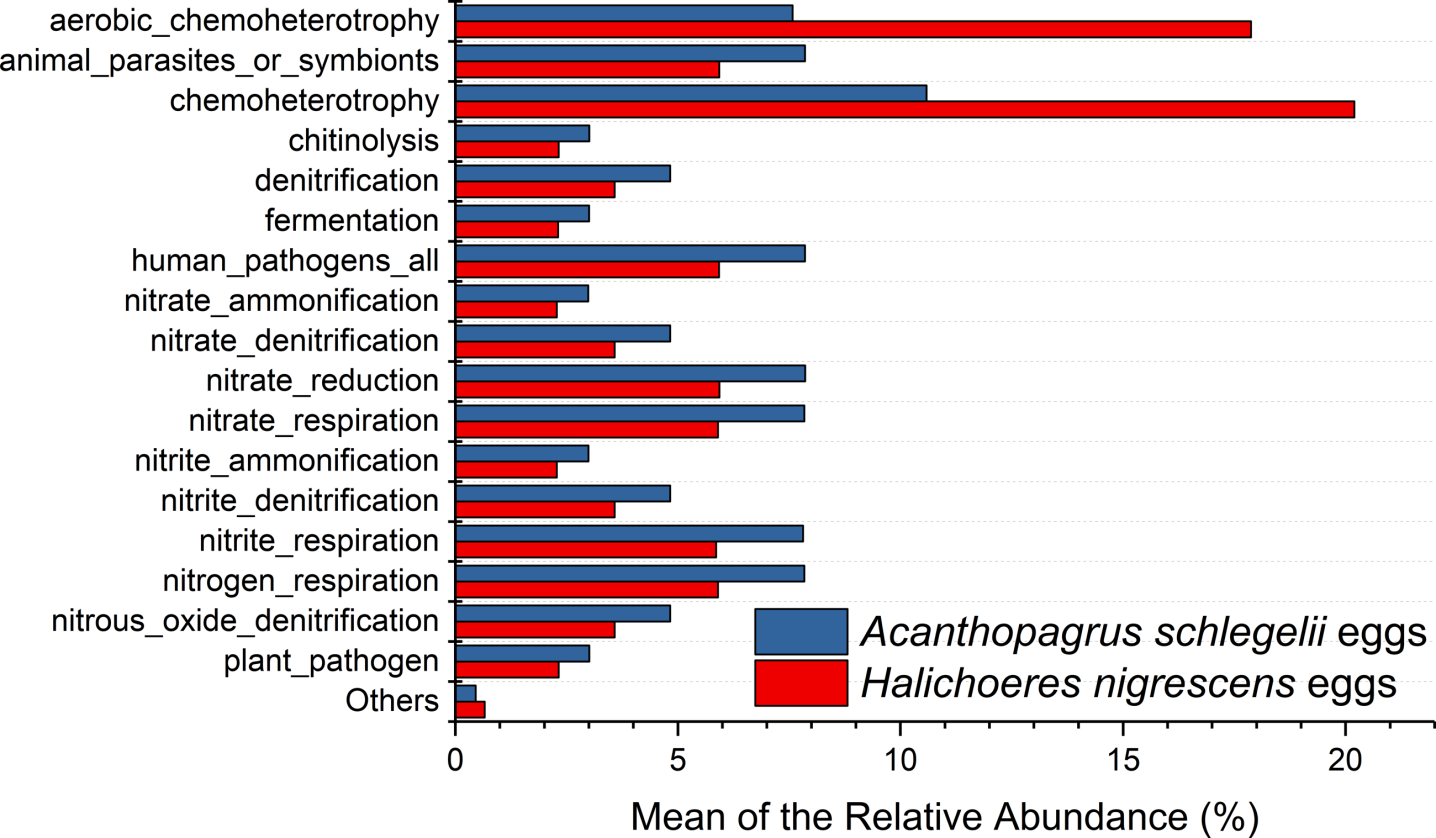
Stacked bar chart showing Mean of the relative abundance of predicted metabolic potential of microbes from *Acanthopagrus schlegelii* and *Halichoeres nigrescens* eggs, as predicted by FAPROTAX .

According to the results of PICRUSt, membrane transport was the most frequently predicted function with an average abundance of 15.8% and 15.2% in *A. schlegelii* and *H. nigrescens,* respectively. Amino acid metabolism (10.7% and 10.7%, respectively), carbohydrate metabolism (8.8% and 8.5%, respectively), replication and repair (5.7% and 5.5%, respectively), and energy metabolism (4.8% and 4.7%, respectively) were also predicted functions for *A. schlegelii* and *H. nigrescens*. The results of the predicted metabolic potential were very similar between the *A. schlegelii* and *H. nigrescens* groups (Fig. 10). The results of FAPROTAX indicated that the microbes of the *A. schlegelii* group were more efficient chemoheterotrophs than *H.nigrescens*, but had a lower nitrogen cycling capacity (Fig.  11).

## Discussion

### Microbial community structure of coral fish eggs

All of the coral fish egg samples for this study were collected within a period of two hours from the same sea area to provide a standardized sample of the microbial community structure. A recent study illustrated that the microbial communities of lumpfish eggs were significantly different between sample sites, such as from intake water, fish tank water, drain pipes, a swab of a tank wall, and the fish themselves (Roalkvam et al., 2019). The Shannon index and Inverse Simpson index were taken after subsampled sequences to determine the microbial alpha diversity indices; the results indicated that the microbial diversity of *A. schlegelii* eggs was higher than that of *H.nigrescens*. The larger egg diameter of *A. schlegelii* provides a greater surface area on which microorganisms might attach and may contribute to the greater microbial diversity found among this species.

The results of PCoA, NMDS, and dissimilarity tests revealed that the microbial community structures differed significantly between the two fish egg species, which may be due to host selection. Many studies have demonstrated that the microbial communities of the fish gut differ by species, such bass, bluegill, catfish, gar, bream, carp, culter, perch, codfish, and snakehead (Tarnecki et al., 2017). However, most of the samples came from cultured adult fish, not wild coral fish eggs. In this study, we showed that the microbial community structures were different between *A. schlegelii* and *H. nigrescens* eggs in the Xuwen Coral Reef National Nature Reserve sea area. The three dominant bacterial taxa (*Pseudomonas*, *Archromobacter*, and *Serratia*) may be common to all samples. 547 OTUs were generated from after the sequences were subsampled and the number of unique OTUs resulting from this subsampling was 218 from the *A. schlegelii* group and 165 from the *H. nigrescens* group. The heatmap results based on the 30 most abundant OTUs showed that the microbial communities from *A. schlegelii* and *H. nigrescens* eggs were different.

### Microbial community composition of the coral fish eggs

Members of Gammaproteobacteria and Betaproteobacteria constituted the major parts of the microbial community composition of the two coral fish eggs, *A. schlegelii* and *H. nigrescens*. Gammaproteobacteria was the most abundant phylum found in Atlantic salmon eggs (Liu et al., 2014) and Alphaproteobacteria was expected to be the most abundant bacteria found in tropical sea water (Gregoracci et al., 2012; Du et al., 2013). Our results showed that the relative abundance of Gammaproteobacteria and Betaproteobacteria from coral fish eggs was 99.1% to 99.7%, indicating that the microbial community composition of fish eggs is distinct from that of the surrounding sea water. Furthermore, *Pseudomonas*, *Achromobacter,* and *Serratia* were the most dominant bacteria associated with *A. schlegelii* and *H. nigrescens* eggs. *Pseudomonas* was the most abundant genus found on brown trout eggs (Wilkins, Fumagalli & Wedekind, 2016); *Vibrio*, *Pseudoalteromonas*, *Pseudomonas,* and *Moraxella* were found on sardine eggs (Míguez & Combarro, 2003); *Pseudomonas*, *Alteromonas*, *Aeromonas*, and *Flavobacterium* were prevalent on cod and halibut eggs (Hansen & Olafsen, 1989). These previously published studies support our findings that *Pseudomonas* is an extremely common bacteria found on fish eggs. However, recent research shows that lumpfish eggs were dominated by *Tenacibaculum*, which is a pathogenic fish bacteria affiliated with the phylum of Bacteroidetes. *Pseudomonas* was not be detected in the microbial composition of lumpfish eggs (Roalkvam et al., 2019). The compositional differences of the main bacterial taxa of *A. schlegelii*, *H. nigrescens*, and lumpfish eggs may due to differences in geography or species, making it important to determine the microbial communities of different fish eggs in specific geographical locations. Many studies have documented that *Pseudomonas*, *Achromobacter,* and *Serratia* are the most common pathogenic and spoilage bacteria in fish (Miller 3rd et al., 1973; Shewan, Hobbs & Hodgeiss, 1960; Baya et al., 1992). *Pseudomonas* and *Achromobacler* may be present in the slime, gills, and intestines of fish (Herbert et al., 1971). A highly pathogenic *Serratia* strain was isolated from the posterior kidney of an adult white perch and the extracellular products (ECP) from the *Serratia* strain were lethal for the fish. This particular strain had strong proteolytic activity that was cytotoxic in fish and homoeothermic cell cultures (Baya et al., 1992). *Serratia* is an opportunistic pathogen and the kidney and spleen are the most vulnerable parts of a fish’s anatomy (Vigneulle & Baudin Laurencin, 1995). *Pseudomonas* and *Achromobacler* were identified as pathogenic and spoilage bacteria in fish in 1960 (Shewan, Hobbs & Hodgeiss, 1960), but since that time no studies have focused on the effects of *Pseudomonas*, *Achromobacler,* and *Serratia* on fish eggs. The results of this study showed that the harmful bacteria, *Pseudomonas*, *Achromobacler* and *Serratia,* may be the reason for eggs failing to hatch or survive to juvenile and adult stages.

### Predicted metabolic potential of microbial communities from coral fish eggs

There have been no studies reported on the microbial communities associated with coral fish eggs. In order to gain more information on the microbial communities, PICRUSt and FAPROTAX were used to predict the metabolic potential of those microorganisms associated with coral fish eggs. PICRUSt analysis supported the prediction made from phylogenetic information about the organisms of the community and the unobserved character states within the community. Gene family abundance (e.g., the metagenome) was predicted in environmental DNA samples for which only the marker gene (e.g., 16S rRNA gene) data was available. FAPROTAX (Functional Annotation of Prokaryotic Taxa), a database that maps prokaryotic clades (e.g., genera, species or subspecies), was used to establish metabolic or other ecologically relevant functions based on the current literature. FAPROTAX includes software for converting taxonomic microbial community profiles (e.g., in the form of an OTU table) into putative functional profiles based on the taxa identified in a sample. Therefore, the results of these predictive functions are theoretical. Our results showed that the most prevalent predicted functions of the microbial communities from coral fish eggs were membrane transport, amino acid metabolism, and nitrogen cycling, which may be related to fish egg respiration. During the development of fish eggs, a large pool of metabolic products are released during respiration and used by the microorganisms that adhere to the egg. However, the predictive functions based on the 16S rRNA gene sequencing data were not included in the results, and other molecular biology techniques including quantitative PCR, metagenomic sequencing, and metatranscriptomic sequencing combined with binning analysis should be applied to reveal more accurate metabolic functions of the microorganisms associated with fish eggs.

## Conclusions

The microbial communities of two coral fish eggs, *A. schlegelii* and *H. nigrescens,* from the Xuwen Coral Reef National Nature Reserve were examined using 16S rRNA gene sequencing. Our results showed that the microbial communities of the eggs from these two fish species is significantly different. Three pathogenic and spoilage bacteria, *Pseudomonas*, *Achromobacter,* and *Serratia,* were the most prevalent bacteria associated with *A. schlegelii* and *H. nigrescens* eggs. This association indicates that a large number of pathogenic and spoilage bacteria associated with fish eggs may prevent them from hatching into larvae and developing into juveniles. The main predicted functions based on 16S rRNA gene sequencing by PICRUSt and FAPROTAX were membrane transport, amino acid metabolism, and nitrogen cycling. Further studies of the microbial communities in different fish egg species from various marine environments should be conducted in the future. In addition, metagenomics, transcriptomics, and proteomics should be studied to determine the functional information of the microorganisms associated with fish eggs.

##  Supplemental Information

10.7717/peerj.8517/supp-1 Figure S1 Rarefaction curve for all samples from *Acanthopagrus schlegelii* (As) and *Halichoeres nigrescens* (Hn) eggs based on sequencing data

10.7717/peerj.8517/supp-2 Supplemental Information 1 Fish eggs identification

## Additional Information and Declarations

### Data Availability

The following information was supplied regarding data availability: Data is available at NCBI: PRJNA560485, and at Figshare: Bai, Shijie (2020): Microbial community of fish eggs on the XuWen coral reef, China. figshare. Dataset. https://doi.org/10.6084/m9.figshare.9746630.v1.
