# Supplementary figures and images for: Microbial communities on fish eggs from Acanthopagrus schlegelii and Halichoeres nigrescens at the XuWen coral reef in the Gulf of Tonkin
Source: PeerJ. 2020 Feb 7;8:e8517. doi: 10.7717/peerj.8517 (PMC7008816; doi:10.7717/peerj.8517)

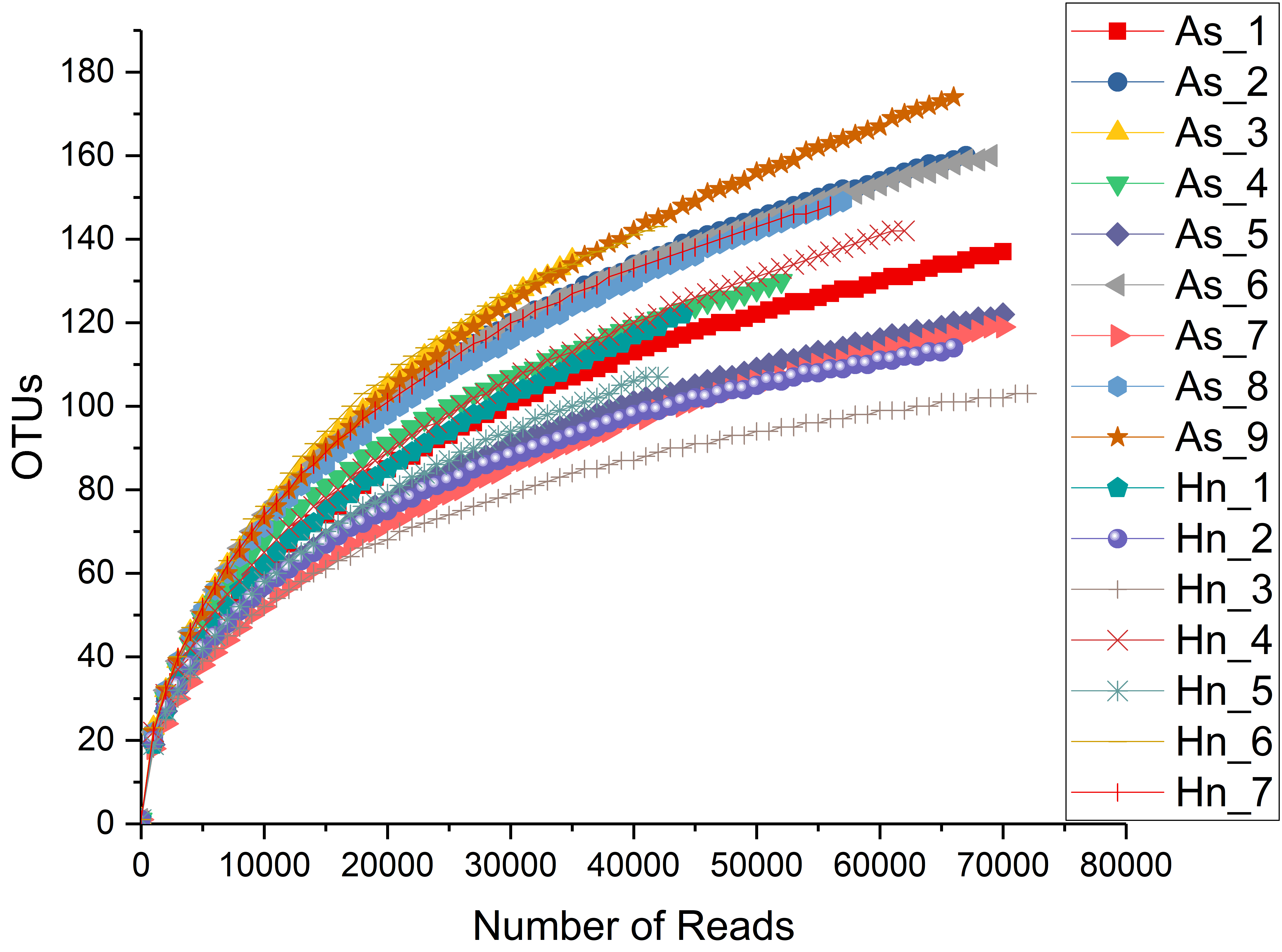

Supplement: Figure S1 [file peerj-08-8517-s001.png]
